# Measuring the Cultural Competence of Latinx Domestic Violence Service Organizations

**DOI:** 10.1177/08862605211025602

**Published:** 2021-06-18

**Authors:** Christopher J. Wretman, Cynthia Fraga Rizo, Jeongsuk Kim, Carolina Alzuru, Deena Fulton, Lisi Martinez Lotz

**Affiliations:** 1 University of North Carolina at Chapel Hill, NC, USA; 2 North Carolina Counts Coalition, Raleigh, NC, USA; 3 North Carolina Department of Health and Human Services, Raleigh, NC, USA; 4 North Carolina Area Health Education Centers, Chapel Hill, NC, USA

**Keywords:** domestic violence, Latinx, cultural competency, measurement, factor analysis

## Abstract

Domestic violence (DV) represents a significant public health concern in the United States, including among Latinx populations. Despite the negative consequences associated with experiencing DV, research has shown that Latinx DV survivors may be less likely than others to utilize important services. One potential barrier is cultural competence (CC) in the provision of services specific to Latinx survivors among DV organizations. Thus, a beneficial addition to the field of DV service provision for such survivors is a better understanding and measurement of CC for this unique population. The exploratory, cross-sectional study herein presents the development and evaluation of a novel instrument for measuring the CC of DV organizations. Exploratory factor analysis was used on a purposive sample of 76 organizations in North Carolina who completed a comprehensive survey on their characteristics, practices, norms, and values. Psychometric results found best support for a 29-item, 4-factor bifactor model with both a general CC factor as well as three sub-factors. The general scale was named “General Cultural Competence,” while the three sub-scales were named “Organizational Values and Procedures,” “Latinx Knowledge and Inclusion,” and “Latinx DV Knowledge.” The final measure also demonstrated convergent validity with key organizational characteristics. Overall, higher CC scores were associated with organizations having more DV services in Spanish, a higher percentage of staff attending CC training, a higher percentage of staff attending Latinx service provision training, and a medium or greater presence in the Latinx community, and a moderate or stronger relationship with the Latinx community. The development of this measure is particularly useful in addressing knowledge gaps regarding the measurement of CC for Latinx DV services. Implications have importance for both the measurement of organizational CC and the scope of the measure’s associations with organizational, provider, and client outcomes.

## Introduction

The phenomenon of physical violence, psychological aggression, sexual violence, and stalking perpetrated by an intimate partner represents a significant public health concern in the United States (U.S.) (Smith et al., 2018). These acts are often collectively referred to as *domestic violence* (DV) among practitioners and service providers in the field (Serrata et al., 2020). Findings from a recent national survey estimate that over one in three women and about one in three men in the United States may experience lifetime DV perpetrated against them by a current or former intimate partner (Smith et al., 2018). These numbers are even more worrisome given that DV is associated with numerous deleterious short- and long-term consequences. In addition to immediate needs related to safety, research has found that DV victimization can lead to physical and mental health problems (Bacchus et al., 2018; Campbell, 2002; Devries et al., 2013; Lagdon et al., 2014) and economic/housing instability (Adams et al., 2012; Pavao et al., 2007) among other concerns. Notably, compared to their male counterparts, female survivors tend to suffer more serious consequences and are more likely to seek DV-related services (Ansara & Hindin, 2010; D’Inverno et al., 2019).

Organizations focused on supporting DV survivors provide an array of services including crisis services, legal and medical advocacy, individual and group counseling, shelter, and others (Macy et al., 2009, 2013, 2018). Historically, DV services were developed and provided using a culturally neutral service delivery approach (Bent-Goodley, 2001, 2005; Lehrner & Allen, 2009). However, researchers and practitioners have been increasingly vocal about the importance of integrating *cultural competence* (CC) in DV service provision given the significant role of race, ethnicity, and culture in understanding and addressing DV (Bent-Goodley, 2005; White et al., 2019). Although organizations across the U.S. are providing DV services to survivors from a variety of different cultural backgrounds, there is limited understanding of how culture may influence service provision and whether such organizations may demonstrate CC.

## Domestic Violence and Latinx Survivors

One prominent population within the United States that constitutes a unique cultural force are those with family roots in Latin/Hispanic America who primarily speak the Spanish language. Varyingly referred to as Hispanic, Latino, Latina, or other names, and collectively referred to herein as *Latinx* people, this population is an important and growing group of U.S. residents that require targeted research attention vis-à-vis DV victimization.

A recent systematic review found that DV is likely common among such people in the US, especially women, with DV prevalence rates among Latinx women ranging from 4% to 80% (Gonzalez et al., 2020). Despite the wide prevalence range reflective of the methodological heterogeneity of studies, these findings overall suggest such women face similar or higher rates of DV compared to their White counterparts (Smith et al., 2017). Compounding these DV experience, research has found that Latinx survivors often experience significant levels of polyvictimization and revictimization (Cuevas et al., 2010, 2012). For example, a national study examining interpersonal victimization among Latinx women found that among those who had experienced one form of victimization, approximately two-thirds reported experiencing more than one incident of interpersonal violence (Cuevas et al., 2012). Moreover, research has also found that the effects of DV may be unique among the Latinx population. Emerging research has found that Latinx women may be disproportionately impacted by physical and mental health outcomes resulting from DV, including persistent health problems, pain, difficulty sleeping, perceived poor health, depression, posttraumatic stress disorder, and anxiety (Bonomi et al., 2009; Cuevas et al., 2010; DiCorcia et al., 2016; Kelly, 2010; Stockman et al., 2015). Also, Latinx women have been found to be at a higher risk of intimate partner homicide compared to White women (Sabina & Swatt, 2015). Altogether, there is good evidence to believe that Latinx women in the United States constitute a particularly vulnerable population affected by DV.

Despite this increased vulnerability, findings have shown that Latinx survivors may be less likely than others to utilize important DV services (Ahmed & McCaw, 2010; Satyen et al., 2018). Among Latinx survivors, those who only speak Spanish and those with no or limited documentation report lower levels of formal help-seeking and service use (Ahmed & McCaw, 2010; Zadnik et al., 2014). Latinx survivors’ underutilization of services has been theoretically and empirically connected to a multitude of help-seeking barriers (O’Neal & Beckman, 2017; Rizo & Macy, 2011). Although some of these barriers are common across many survivor groups, others are likely either unique or more pronounced for survivors from racial/ethnic groups that have been marginalized (Rizo & Macy, 2011; Robinson et al., 2020). Research suggests that such survivors, broadly, may experience culturally-based barriers to DV service receipt related to language, social isolation, and gender norms (O’Neal & Beckman, 2017; Parson et al., 2016; Postmus et al., 2014; Reina et al., 2014; Rizo & Macy, 2011). Also, such DV survivors may also face disproportionate socioeconomic barriers related to educational attainment, poverty, and distribution of resources (O’Neal & Beckman, 2017; Reina & Lohman, 2015; Vidales, 2010). Latinx survivors, specifically, may also experience barriers related to anti-immigrant and anti-Latinx policies, beliefs, and practices, such as fear of deportation and discriminatory treatment (O’Neal & Beckman, 2017; Parson et al., 2016; Postmus et al., 2014; Reina & Lohman, 2015; Rizo & Macy, 2011).

Overall, the lack of culturally competent services and negative prior help-seeking experiences are identified as barriers to Latinx survivors’ DV-related help-seeking (Flicker et al., 2011; Rizo & Macy, 2011). In particular, research has emphasized the importance of culture in Latinx survivors’ DV experiences as well as their experiences seeking and receiving services (O’Neal & Beckman, 2017; Postmus et al., 2014; Serrata et al., 2020).

## Cultural Competence and DV Services for Latinx Survivors

In response to growing research on the unique experiences and needs of Latinx DV survivors, both researchers and practitioners are calling for more culturally competent services to increase access, help-seeking, and service engagement (Alvarez & Fedock, 2018; Alvarez et al., 2018, 2016; Robinson et al., 2020). DV organizations and service providers are being urged to develop a nuanced understanding of Latinx culture and identity to better understand the needs of these survivors (Serrata et al., 2020; Silva-Martínez & Murty, 2011). Recommendations include accounting for cultural barriers and incorporating cultural factors into services and service delivery (Parra-Cardona et al., 2013; Reina et al., 2014; Serrata et al., 2020). Culturally competent and affirming practices highlighted in the literature include hiring Latinx and Spanish-speaking staff, encouraging English-speaking staff to learn key phrases in Spanish, ensuring resources and materials are available in Spanish, engaging in culturally specific outreach to increase awareness, and promoting cultural traditions among others (O’Neal & Beckman, 2017; Parson et al., 2016; Serrata et al., 2020). Such practices have been found to enhance Latinx survivors’ well-being over and above trauma-informed practices (Serrata et al., 2020).

Given that culturally competent practice requires organizational support and infrastructure (Balcazar et al., 2009; Sharifi et al., 2019), it is necessary to understand the CC of organizations providing DV services to Latinx survivors. Organizational CC is generally concerned with an organization’s values, policies and procedures, planning and evaluation, communication, human resources, community and client engagement, services, and organizational resources (Harper et al., 2006; Zeitlin Schudrich, 2014). Limited research has examined the CC of DV organizations and practices, particularly as this relates to serving Latinx survivors (Lucero et al., 2020). One challenge to the advancement of such research is the lack of tailored instruments for measuring the CC of organizations providing DV services to Latinx survivors. Despite the existence of general organizational CC instruments, these instruments have undergone relatively little psychometric testing (Guerrero & Andrews, 2011)—and a review of the literature was unable to identify any that had been tested with DV organizations. Further, growing research highlights the importance of tailoring such instruments to specific client groups given that organizational CC can vary by culture, race, and ethnicity (Siegel et al., 2011).

An instrument specifically developed to assess the CC of organizations providing DV services to Latinx survivors could benefit the field in multiple ways. Organizations providing DV services to Latinx survivors could use such an instrument to monitor and improve the CC of their organization, service delivery approaches, and specific services. Researchers could also use the instrument to examine the CC of organizations providing such services nationally, as well as the malleable factors associated with enhancing organizational CC. A better understanding of the factors associated with organizational CC among organizations serving Latinx survivors could inform the development of interventions aimed to increasing the cultural appropriateness of such organizations.

## Current Study

To advance research and practice focused on understanding and enhancing the CC of DV service provision for Latinx survivors, the current study presents the development and preliminary evaluation of an instrument for measuring the CC of organizations providing DV services for Latinx survivors. Thus, this exploratory study sought to address knowledge gaps regarding the measurement of CC among such organizations. A well-validated measure is critical to not only understanding the CC of organizations providing DV services, but also to enhancing the CC of such organizations (Zeitlin Schudrich, 2014). Therefore, the overall goal of this exploratory, cross-sectional study was to develop a psychometrically valid measure of organizational CC using exploratory factor analysis (EFA) to facilitate the measurement of DV service provision for Latinx survivors among organizations in North Carolina in the United States. The study featured the following aims: (a) to evaluate the *factorial validity* of a scale for use in Latinx DV service provision, and (b) to evaluate the *construct validity* of the scale relative to organizational characteristics. Thus, this study sought to both establish the measure’s validity and then understand how it might differentiate among organizations.

## Methods

### Sample

The sample comprised organizations that identified as either being (a) a DV-specific organization or (b) a Latinx organization that served clients presenting with DV-related concerns. All organizations were located in North Carolina—the 9th largest state in the United States with almost 1,000,000 Latinx residents. These organizations were participants in a statewide study of DV service provision for Latinx survivors. The overall study aimed to better understand DV service provision for Latinx survivors, including (a) service gaps, (b) program needs, and (c) challenges experienced in providing culturally competent services to inform trainings and technical assistance, policy, and funding. The study was conducted jointly by a research team at the University of North Carolina at Chapel Hill (UNC-Chapel Hill) in collaboration primarily with the North Carolina Coalition Against Domestic Violence (NCCADV)—a key state-level DV organization in North Carolina. All research procedures were approved by UNC-Chapel Hill.

The study’s sampling frame was constructed in two phases. First, the UNC-Chapel Hill team worked with the NCCADV to compile a full list of DV organizations within the state. Second, to identify organizations that primarily provide culturally specific services to Latinx populations, the UNC-Chapel Hill team searched online and emailed individual organizations to confirm their service provision information. In total, 99 organizations were contacted. Participating organizations were eligible for one of three $100 gift cards.

### Measures

Data for analysis of the organizations’ characteristics and practices were collected via a purposive, study-specific survey. The survey featured approximately 260 open- and closed-ended questions in total over six broad domains related to (a) community characteristics, (b) organization characteristics, (c) service delivery, (d) organizational CC, (e) barriers to service, and (f) respondent characteristics. Development of the survey was conducted according to best practices in measurement development (DeVellis, 2012). Specific steps included (a) conceptualization of key constructs, (b) development of an initial item pool, (c) determination of formatting, (d) initial expert review, (e) pilot testing, and (f) optimization and finalization. Initial development of items was determined by the research team’s expertise and past work related to DV service provision, narrative reviews of literature and existing measures, and consultation with the NCCADV. Experts involved in review of the survey included staff at the NCCADV as well as other selected North Carolina DV service providers.

There were 32 questions in the survey related specifically to organizational CC of DV service provision for Latinx survivors. These questions were both adapted from external sources and developed internally by the research team. External sources that inspired the items included (a) the NCCADV’s internal LGBTQ DV assessment instrument, (b) the *Cultural Competence Self-Assessment Questionnaire* (Mason, 1995), (c) the *Cultural Competence Assessment Instrument* (Balcazar et al., 2009), and (d) the *Cultural Competence Assessment Scale* (Siegel et al., 2011). The final 32 items covered a broad array of topics related to organization (a) characteristics, (b) practices, (c) norms, and (d) values. Questions were primarily Latinx-specific (*n* = 30, 93.8%; e.g., “Our organization prepares new staff to work with Latinx DV survivors”), with some additional generalized items (*n* = 2, 6.3%; e.g., “Our organization staff routinely discuss barriers to working across cultures”). Within the larger survey, these CC questions were demarcated within a box and preceded by a prompt asking respondents to “Please answer the following by marking the answer box that best reflects your level of agreement with each statement.” Response options comprised “strongly disagree” (1), “disagree” (2), “neither agree/disagree” (3), “agree” (4), and “strongly agree” (5). As presented in the original survey completed, the 32 questions had a collective Flesch reading ease of 32.2, indicative of “difficult” readability (Flesch, 1948)—a level appropriate to college graduates such as those working in the sample of organizations.

One respondent, typically the organization’s Executive Director, answered the survey on behalf of their entire organization. Respondents were offered the option to complete the survey electronically via Qualtrics (Qualtrics, Provo, UT), by paper form delivered and returned via mail, or by telephone with the assistance of a trained research assistant. The entire survey took approximately 60-75 minutes to complete. Data collection occurred from 2015 to 2016.

### Analysis

EFA was chosen as the primary analytic approach as a psychometric data reduction method that explores variability among correlated observed items (i.e., the survey questions) in a measure within the context of specifying a parsimonious underlying latent variable. The analytic plan included five sequential phases. All non-EFA analyses were conducted using Stata 16.1 (StataCorp, College Station, TX) and all EFA-specific analyses were conducted in Mplus 7.3 (Muthén & Muthén, Los Angeles, CA). A statistical significance level of *p* < .05 (two-sided) was used throughout.

First, select organization characteristics were summarized using appropriate univariate statistics (e.g., frequency [*n*], proportion [%], mean [*M*], standard deviation [*SD*]) to describe (a) the nature of the sample and (b) targets for subsequent construct validity analyses. There were 15 total characteristics, with three characteristics for each *post hoc* determined domain of (a) service delivery and location, (b) staff numbers and characteristics, (c) staff training, (d) client profile, and (e) Latinx outreach.

Second, preliminary diagnostic tests were conducted as (a) omnibus tests of all 32 CC items jointly and (b) individual tests of each CC item. The primary goal of such tests was to reduce, if possible, the starting item pool to a more parsimonious set. A secondary goal was to better understand item characteristics and the hypothesized potential latent structure of the items. Omnibus tests were specified to focus primarily on analysis of communalities (*h*^2^), or the total amount of variance explained by the hypothesized CC latent variable. A *h*^2^ ≥ 0.70 criterion was set for inclusion in further analyses due to research consistently showing that high communalities are vital to acceptable fit and factor recovery when conducting EFA with small samples such as was the case herein (de Winter et al., 2009; Mundfrom et al., 2005; Preacher & MacCallum, 2002). An additional omnibus check was Bartlett’s test of sphericity, with a statistically significant *Χ*^2^ value sought. Kaiser-Meyer-Olkin (KMO) tests of sampling adequacy were specified both at an omnibus level and for each item, with KMO ≥ 0.80 being considered “meritorious” and desirable. Individual items’ observation-level missingness was also calculated.

**Figure 1.**
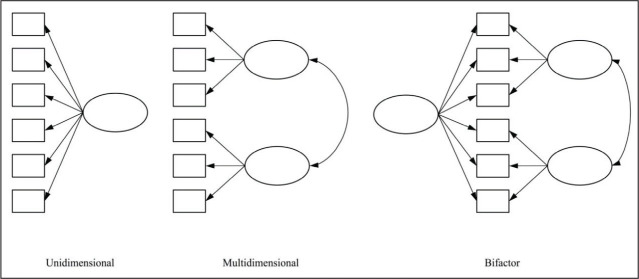
Unidimensional, multidimensional, and bifactor latent factor models.

Third, factorial validity was tested via EFA on the total sample of 76 observations using a strategy that sought to compare competing solutions with varying (a) dimensionality and (b) factor number, essentially comprising sensitivity analyses of the factorial validity. Given the desire to explore a range of model solutions, no pre-EFA tests (e.g., Horn’s parallel analysis) were conducted to determine an exact number of factors to be extracted. The approaches included (a) unidimensional, (b) multidimensional, and (c) bifactor models to provide a comprehensive exploration of the potential underlying latent structure of the hypothesized CC variable. These models can be visualized conceptually in Figure 1. The bifactor models, in particular, represented a novel approach within DV measurement. These models, which posit a general latent factor alongside distinct subfactors, have heretofore been underused in violence psychometric research but have become a powerful choice for EFA analyses in other fields (Bryan & Harris, 2019; Gracia et al., 2020; Mancini et al., 2019). Overall, the EFA analyses held a strong desire to keep the number of factors low due to (a) substantive concerns regarding applicability in real world settings of DV service provision, and (b) methodological concerns regarding model parsimony with small samples.

Each approach used principal axis factoring with an oblique geomin rotation using Mplus’ weighted least squares estimator as appropriate for the ordinal nature of the items. Within each approach, models were assessed for (a) overall model fit and (b) individual item appropriateness using *a priori* specified criteria. Model fit was compared using a set of four estimates comprising (a) the root mean square error of approximation (RMSEA; point estimate and 90% confidence interval [CI] ≤ 0.08 = adequate, ≤ 0.06 = good), (b) the comparative fit index (CFI; ≥ 0.90 = adequate, ≥ 0.95 = good), (c) the Tucker-Lewis index (TLI; ≥ 0.90 = adequate, ≥ 0.95 = good), and (d) the standardized root mean square residual (SRMR; ≤ 0.08 = acceptable, ≤ 0.06 = good). All indices and criteria were chosen based on a review of expert recommendations (Browne & Cudeck, 1993; Hu & Bentler, 1999; West et al., 2012). Each model’s *Χ*^2^ statistic and was reported for intermodel comparison, but was not used as a criterion for final model selection. Individual items were assessed based on their factor loadings (λ), with a rule set that an item must feature at least one λ ≥ 0.50 on ≥ 1 factor. Items not meeting this criterion were deleted iteratively starting with the smallest maximum λ.

Fourth, proceeding from selection of a final model solution, the total scores were created and described. Aggregate scores were created by calculating the unweighted mean of all items for each factor. Internal consistency reliability estimates in the form of Cronbach’s α were calculated with a target reliability level of α ≥ 0.70, equivalent to “acceptable” or greater, specified based on recommendations (Nunnally, 1978). Next, total mean interitem correlations (*r*) were calculated with estimates sought that were (a) positive, (b) approximately medium (*r* ≥ 0.30), and (c) significant. Identical criteria were then used to evaluate interscale Spearman’s rank-order correlations among all scales. Finally, Flesch reading ease scores were calculated to determine if the final scales corresponded to levels considered approachable for individuals with either “college” (50.0-30.0) or “college graduate” (30.0-10.0) educational levels as appropriate for the sample (Flesch, 1948).

Fifth, construct validity was established using (a) Spearman’s rank-order correlations for continuous organization characteristics and (b) point-biserial correlations for categorical characteristics. Characteristics to be included were *a priori* determined to be all 15 used to summarize the sample of organizations (see above). Convergent validity would be determined with (a) consistently positive, (b) approximately medium (*r* ≥ 0.30), and (c) significant correlations across factors. Divergent validity would be determined by (a) low (*r* < 0.30) and (b) nonsignificant correlations.

## Results

### Organization Characteristics

Of the 99 organizations contacted, a total of 82 participated in the survey in some form. Among those, two exclusion criteria were applied to remove participants that either (a) reported not serving clients with DV/SA issues in the previous year (*n* = 3) or (b) did not answer any of the CC items (*n* = 3). The final analytic sample was 76, making for a final response rate of 76.8% of the total of 99 that were recruited.

Respondents (Table 1) indicated that two-thirds of the organizations were dual DV/SA organizations (*n* = 50; 66%), with the others being standalone DV organizations (*n* = 14; 18%) or culturally specific Latinx organizations (*n* = 12; 16%). Organizations were small, with a mean number of full-time staff of 10.0 (*SD* = 9.8) and a mean number of part-time staff of 6.4 (*SD* = 7.1). Over two-thirds (*n* = 51; 67%) had at least one Spanish-speaking Latinx staff member. Although the majority of staff (*M* = 79%; *SD* = 33.6) had attended DV training of some type, only 39% (*SD* = 32.2) had attended any Latinx service provision training. The mean number of clients served in the previous year (i.e., 2014) was 850.5 (*SD* = 1,333.6), an indication alongside the relatively high proportion of multicounty service (*n* = 31; 41%), that the organizations had a generally wide scope of operation. Latinx clients were unsurprisingly high given North Carolina’s burgeoning population of Latinx residents, with on average 25% (*SD* = 28.9) and 24% (*SD* = 29.9) of all clients being Latinx or primarily speakers of Spanish, respectively. Half of the organizations reported a medium-to-high presence (*n* = 37; 50%) and approximately two-thirds reported a moderate-to-strong relationship (*n* = 47; 65%) with their Latinx community.

**Table 1.** Agency Characteristics (*N* = 76).

Characteristics	*N* (%) or Mean (*SD*)
Type	
Stand-alone DV agency = Yes	14 (18.4)
Dual DV and SA agency = Yes	50 (65.8)
Culturally-specific agency serving the Latinx community = Yes	12 (15.8)
Service delivery and location	
# of DV services provided in Spanish (0-17)^1^	7.82 (4.43)
Serves more than one county = Yes	31 (40.8)
Serves only rural locations = Yes	48 (64.0)
Staff numbers and characteristics	
# of full-time staff (0–)	9.95 (9.79)
# of part-time staff (0–)	6.37 (7.11)
Has Latinx Spanish-speaking staff = Yes	51 (67.1)
Staff training	
% of staff members attended DV training (0.0–)	78.56 (33.59)
% of staff members attended cultural competence training (0.0–)	53.62 (32.83)
% of staff members attended Latinx service provision training (0.0–)	39.19 (32.22)
Client profile	
# of clients served in the previous year (0–)	850.48 (1,333.61)
% of clients served in the previous year that were Latinx (0.0–)	25.30 (28.87)
% of clients served in the previous year that spoke primarily Spanish (0.0–)	24.34 (29.93)
Latinx outreach	
Has a medium-to-high presence in the Latinx community = Yes	37 (50.0)
Has a moderate-to-strong relationship with the Latinx community = Yes	47 (65.3)
# of outreach activities to address DV in the Latinx community (0-14)^1^	5.84 (3.31)

*Note*. DV = domestic violence; SA = sexual assault. ^1^Composite of individual items. Due to missing data, response frequencies range from 69 to 76 among characteristics. Data collected August 2015 through January 2016.

### Factorial Validity

*Item diagnostics*. The omnibus test of the 32-item set revealed that two items should be dropped due to low communalities. The first item related to organizations’ use of a “written cultural competence plan” for serving Latinx DV survivors (*h*^2^ = 0.60), while the second assessed if organizations’ Boards included “representative(s) from the Latinx community” (*h*^2^ = 0.60). After removing these two items, the remaining set of *k* = 30 demonstrated good communalities (*h*^2^_Mean_ = 0.83). Additionally, Bartlett’s test rejected the null hypothesis that the correlation matrix is equal to an identity matrix, indicating that the observed items were likely indicators of an underlying latent construct (*p* < .001). The overall 30-item KMO value was acceptable at 0.87 and individual item KMO values ranged from 0.75 to 0.95, with only three being less than 0.80. Of the 30 items, fourteen (46.7%) had no missing values, eight (26.7%) had one, and four (13.3%) each had two or three missing values. No missing data were imputed in subsequent analyses.

Approach 1: Unidimensional model. For the unidimensional model specifying one general factor (Table 2), applying the λ ≥ 0.50 criterion resulted in a model with 29 items. The model’s fit was unacceptable: Χ^2^ = 1,111.85, p < .001; RMSEA = 0.16 (90% CI = 0.15, 0.17); CFI = 0.84; TLI = 0.83; SRMR = 0.14.

**Table 2.** Exploratory Factor Analysis Models of and Organizational Cultural Competence Measure for Latinx DV Service Provision.

Characteristics	Approach #1: Unidimensional	Approach #2: Multidimensional			Approach #3: Bifactor		
		2-Factor Model	3-Factor Model	4-Factor Model	3-Factor Model^1^	4-Factor Model^1^	5-Factor Model^1^
Specification							
Dropped items	1	3	5	7	1	1	2
Fit							
* Χ*^2^ (*p*)	1111.852 (<.001)	760.126 (<.001)	493.428 (<.001)	329.780 (<.001)	639.100 (<.001)	490.978 (<.001)	394.101 (<.001)
RMSEA (90% CI)	0.160 (0.149, 0.171)	0.143 (0.130, 0.155)	0.124 (0.109, 0.139)	0.113 (0.095, 0.131)	0.114 (0.101, 0.127)	**0.093 (0.078, 0.107)**	**0.088 (0.071, 0.104)**
CFI	0.843	**0.900**	**0.933**	**0.962**	**0.932**	**0.958**	**0.968**
TLI	0.831	0.882	**0.912**	**0.943**	**0.915**	**0.943**	**0.951**
SRMR	0.138	0.101	**0.070**	**0.050**	**0.073**	**0.054**	**0.046**

*Note*. DV = domestic violence. # of items = 30. ^1^Includes general factor. Analyses conducted in Mplus 7.3. Bolded values represent approximately “acceptable” or better fit estimates. Data collected August 2015 through January 2016.

*Approach 2: Multidimensional models.* For the multidimensional models (Table 2), the 2-factor model resulted in 27 items being modeled after applying the λ ≥ 0.50 criterion and demonstrated “acceptable” fit according to one of four estimates: *Χ*^2^ = 760.13, *p* < .001; RMSEA = 0.14 (90% CI = 0.13, 0.16); CFI = 0.90; TLI = 0.88; SRMR = 0.10. Meanwhile, the 3-factor multidimensional model featured 25 items and demonstrated “acceptable” fit on three of four estimates: *Χ*^2^ = 493.43, *p* < .001; RMSEA = 0.12 (90% CI = 0.11, 0.14); CFI = 0.93; TLI = 0.91; SRMR = 0.07. Finally, the 4-factor multidimensional model featured 23 items, and demonstrated “acceptable” or “good” fit on three of four estimates: *Χ*^2^ = 329.78, *p* < .001; RMSEA = 0.11 (90% CI = 0.10, 0.13); CFI = 0.96; TLI = 0.94; SRMR = 0.05.

*Approach 3: Bifactor models.* For the bifactor approach (Table 2), the 3-factor model (1 general + 2 correlated subfactors) featured 29 items and demonstrated “acceptable” fit according to three estimates: *Χ*^2^ = 639.10, *p* < .001; RMSEA = 0.11 (90% CI = 0.10, 0.13); CFI = 0.93; TLI = 0.92; SRMR = 0.07. Meanwhile, the 4-factor model (1 general + 3 correlated subfactors) was also estimated with 29 items, and demonstrated “acceptable” or “good” fit on three of four estimates: *Χ*^2^ = 490.98, *p* < .001; RMSEA = 0.09 (90% CI = 0.08, 0.11); CFI = 0.96; TLI = 0.94; SRMR = 0.05. Finally, the 5-factor bifactor model (1 general + 4 correlated subfactors) featured 28 items and “acceptable” or “good” fit on three of four estimates: *Χ*^2^ = 394.10, *p* < .001; RMSEA = 0.09 (90% CI = 0.07, 0.10); CFI = 0.97; TLI = 0.95; SRMR = 0.05.

### Measure Summary

The final chosen model was the 4-factor bifactor model (Table 3). Although this solution featured slightly worse fit compared with the 5-factor bifactor model, it was chosen due to parsimony and face validity of the resultant three subscales. This model’s suboptimal RMSEA values were not seen as a major limitation given the exploratory nature of the work. The study team named the general scale for this solution “General Cultural Competence” (GCC), while the three subscales were named “Organizational Values and Procedures” (OVP), “Latinx Knowledge and Inclusion” (LKI), and “Latinx DV Knowledge” (LDK).

**Table 3.** Item Characteristics of an Organizational Cultural Competence Measure for Latinx DV Service Provision.

Organizational Cultural Competence Items	Factor Loadings				Mean(*SD*)
	General	#1 (OVP)	#2 (LKI)	#3 (LDK)	
1. Our agency talks about Latinx issues in general at an organizational level.^1^	0.766				3.87 (0.91)
2. Our agency talks about Latinx DV survivors and the unique needs of these survivors at an organizational level.^1^	0.743				3.67 (1.14)
3. Our agency’s programmatic goals reflect the ways our work impacts Latinx DV survivors and communities.^1^	0.707				3.42 (1.01)
4. Anti-Latinx bias is a part of regular discussions or trainings at staff and/or board meetings.^1^	0.636				2.83 (1.15)
5. Anti-immigrant bias is a part of regular discussions or trainings at staff and/or board meetings.^1^	0.615				2.79 (1.12)
6. Our agency staff routinely discuss barriers to working across cultures.^2^	0.679				3.62 (1.01)
7. Our agency staff engage in self-reflection regarding working with Latinx DV survivors.	0.727				3.46 (0.97)
8. Our agency events are mindful and inclusive of the Latinx community.^1^	0.719		0.430		3.72 (0.97)
9. Our agency participates in cultural events related to the Latinx community in our service area.^2^	0.699		0.365		3.55 (1.05)
10. Our agency is accountable to the Latinx community in our service area.^1^	0.731				3.82 (0.96)
11. Our agency formally or informally seeks feedback from Latinx DV survivors regarding the services we provide. ^1^	0.674				3.67 (1.04)
12. Cultural competence specific to working with Latinx DV survivors is included in our agency’s mission statement, policies, and procedures.^3^	0.627	0.338			3.08 (1.04)
13. Our agency’s budget reflects a commitment to Latinx DV survivors.^1^	0.765				3.17 (1.07)
14. Our agency is committed to hiring and retaining Latinx direct service staff.^4^	0.661				4.00 (0.99)
15. Our agency prepares new staff to work with Latinx DV survivors.^2^	0.768				3.49 (1.03)
16. Pictures, posters, printed materials, and toys at our agency reflect Latinx survivors’ culture and ethnic backgrounds.^3^	0.750		0.324		3.57 (1.05)
17. Most agency staff are able to describe the cultural strengths of the Latinx community.^2^	0.722		0.592		3.49 (0.99)
18. Most agency staff know the prevailing beliefs, customs, norms, and values of the Latinx community.^2^	0.703		0.637		3.46 (0.99)
19. Most agency staff know how DV is viewed by the Latinx community.^2^	0.787			0.388	3.58 (0.96)
20. Most agency staff are knowledgeable about the needs of Latinx DV survivors.	0.815			0.380	3.58 (0.95)
21. Most agency staff are knowledgeable about how DV is typically addressed in Latin American countries.	0.751			0.425	3.32 (0.99)
22. Most agency staff are aware of the documentation policies of all the agencies to which we refer Latinx DV survivors.	0.745			0.586	3.33 (1.00)
23. Most agency staff are aware of the cultural competency of the agencies to which Latinx DV survivors are referred.	0.738			0.585	3.41 (0.90)
24. Our agency uses culturally-specific treatment approaches when working with Latinx DV survivors. ^2^	0.677				3.49 (0.95)
25. Our agency allows Latinx DV survivors to bring and include their family in the services we provide.	0.552	0.614			3.97 (0.95)
26. Our agency staff discuss the parameters of confidentiality related to immigration and deportation with all of our Latinx DV survivors.	0.772	0.329			3.61 (1.06)
27. Our agency purposefully incorporates positive aspects of Latinx culture in the services we provide to Latinx DV survivors.	0.884	0.343			3.67 (0.96)
28. As a whole, agency staff respect the cultural beliefs, norms, and practices of our Latinx DV survivors.	0.629	0.445			4.05 (0.88)
29. Some of our services targeting Latinx DV survivors are provided in community settings.	0.610	0.348			3.42 (1.05)

*Note*. DV = domestic violence; *SD* = standard deviation; OVP = Organizational Values and Procedures; LKI = Latinx Knowledge and Inclusion; LDK = Latinx DV Knowledge. Measure = bifactor exploratory factor analysis model with 1 general factor + 3 subfactors; higher values indicated greater cultural competence. Only factor loadings ≥ .30, significant (*p* < .05), and positive are shown. Due to missing data, response frequencies range from 73 to 76 among items. Response options: “strongly disagree” (1), “disagree” (2), “neither agree/disagree” (3), “agree” (4), “strongly agree” (5). Flesch reading ease scores: General = 34.3, #1 = 27.3, #2 = 33.9, #3 = 42.4. Data collected August 2015 through January 2016. Developed in 2020. ^1^ Items adapted from the North Carolina Coalition Against Domestic Violence’s LGBTQ Domestic Violence Assessment Instrument. ^2^Items adapted from the Cultural Competence Self-Assessment Questionnaire (Mason, 1995). ^3^Items adapted from the Cultural Competence Assessment Instrument (Balcazar et al., 2009). ^4^Items adapted from the Cultural Competence Assessment Scale (Siegel et al., 2011).

The GCC general scale had a total mean score of 3.52 (*SD* = 0.68), with an internal consistency α = 0.96 and a mean interitem correlation of *r* = 0.44. Among the GCC’s individual items (Table 3), mean item scores ranged from 2.79 (*SD* = 1.12) to 4.05 (*SD* = 0.88). The three subscales had similar mean scores (*M*_OVP_ = 3.64, *SD*_OVP_ = 0.78; *M*_LKI_ = 3.56, *SD*_LKI_ = 0.86; *M*_LDK_ = 3.45, *SD*_LDK_ = 0.84), internal consistency (α_OVP_ = 0.88; α_LKI_ = 0.91; α_LDK_ = 0.93), and mean interitem correlations (*r*_OVP_ = 0.55; *r*_LKI_ = 0.66; *r*_LDK_ = 0.72). The six interscale correlations were all significant: *r*_GCC-OVP_ = 0.85, *r*_GCC-LKI_ = 0.73, *r*_GCC-CA_ = 0.81, *r*_OVP-LKI_ = 0.58, *r*_OVP-LDK_ = 0.64, and *r*_LKI-LDK_ = 0.56. The 29 items for the GCC general scale had a Flesch reading ease score of 34.3, with the three subscales having scores of 27.3, 33.9, and 42.4, respectively. The final *n*:*k*, or observation-to-item ratio, was thus 76:29, or 2.6:1, representing a slight increase as desired from the original ratio of 76:32 (i.e., 2.4:1).

### Construct Validity

The general scale and subscales all demonstrated construct validity vis-à-vis their associations with organizations’ characteristics. In total, nine characteristics had ≥ 1 positive and significant correlations with ≥ 1 scale, totaling 24 such correlations out of 60 possible (40.0%; 0.23 ≥ *r* ≤ 0.47). The GCC scale was significantly associated with seven of the 15 characteristics, while the three OVP, LKI, and LDK subscales had five, eight, and four significant correlations, respectively (not pictured).

Table 4 organizes the 11 characteristics with the most consistent (≥ 3 of 4 scales) relationships into *post hoc* determined convergent and divergent domains. Overall, higher CC scores were associated with organizations having (a) more DV services in Spanish, (b) a higher percentage of staff attending CC training, (c) a higher percentage of staff attending Latinx service provision training, (d) a medium or greater presence in the Latinx community, and (e) a moderate or stronger relationship with the Latinx community. Six characteristics were not significantly associated with any of the four scales (range: −0.14 to 0.20). Overall, higher CC was not associated with (a) serving more than one county, (b) serving only rural locations, (c) having more full-time staff, (d) having more part-time staff, (e) having a higher percentage of staff attend general DV training, or (f) having more total clients.

**Table 4.** Correlations of an Organizational Cultural Competence Measure for Latinx DV Service Provision and Agency Characteristics.

Characteristics	Organizational Cultural Competence Factors*r* (*p*)			
	General	#1 (OVP)	#2 (LKI)	#3 (LDK)
Convergent				
# of DV services provided in Spanish (0-17)^1^	**0.41 (<.001)**	**0.33 (.003)**	0.21 (.065)	**0.32 (.005)**
% of staff members attended cultural competence training (0.0–)	**0.35 (.004)**	0.23 (.059)	**0.29 (.018)**	**0.37 (.002)**
% of staff members attended Latinx service provision training (0.0–)	**0.37 (.002)**	**0.30 (.011)**	**0.32 (.007)**	**0.33 (.006)**
Has a medium-to-high presence in the Latinx community = Yes	**0.31 (.007)**	**0.28 (.015)**	**0.36 (.002)**	0.15 (.20)
Has a moderate-to-strong relationship with the Latinx community = Yes	**0.35 (.003)**	**0.23 (.047)**	**0.37 (.001)**	0.17 (.16)
Discriminant				
Serves more than one county = Yes	0.10 (.38)	0.11 (.35)	0.06 (.62)	0.08 (.48)
Serves only rural locations = Yes	−0.08 (.51)	−0.01 (.94)	−0.14 (.22)	−0.003 (.98)
# of full-time staff (0–)	0.07 (.57)	0.03 (.77)	−0.07 (.54)	−0.004 (.98)
# of part-time staff (0–)	0.08 (.51)	−0.02 (.86)	−0.03 (.77)	−0.002 (.99)
% of staff members attended DV training (0.0–)	0.10 (.42)	0.05 (.71)	−0.09 (.46)	0.10 (.40)
# of clients served in the previous year (0–)	0.17 (.15)	0.11 (.35)	0.11 (.38)	0.20 (.090)

*Note*. DV = domestic violence; OVP = Organizational Values and Procedures; LKI = Latinx Knowledge and Inclusion; LDK = Latinx DV Knowledge. ^1^Composite of individual items. Measure = bifactor exploratory factor analysis model with 1 general factor + 3 subfactors; higher values indicated greater cultural competence. Due to missing data, response frequencies range from 68 to 76 among characteristics. *p* values test association of agency characteristics with factor scores using Spearman’s rank correlation (continuous) or point-biserial correlation (categorical); values <.05 bolded. Data collected August 2015 through January 2016.

## Discussion

This exploratory, cross-sectional study used EFA to develop a psychometrically valid measure of CC for Latinx DV service provision using data on 76 organizations in North Carolina in the United States. Taking inspiration from psychometric research on other measures that has brought attention to the utility of comprehensive testing of multiple competing factorial structures (Ebesutani et al., 2012; Mancini et al., 2019; Reise et al., 2010), the analytic approach compared unidimensional, multidimensional, and bifactor EFA approaches across seven individual models. Results demonstrated substantive and methodological preference for a 29-item, 4-factor bifactor EFA model with both a general CC factor/scale as well as three subfactors/scales. In addition to addressing a knowledge gap regarding the measurement of CC for Latinx DV service provision, the current study contributes to the limited psychometric testing of instruments for measuring organizational CC (Zeitlin Schudrich, 2014). Implications from the findings of this work have importance for both (a) the measurement of organizational CC and (b) the scope of the measure’s associations with organizational, provider, and client outcomes.

### Measurement Structure

At a broad level, this study demonstrated that it is possible to validly measure CC among DV service providers serving Latinx survivors—seemingly the first examination of its kind into this important consideration for DV service delivery. What remains inconclusive, however, is exactly how that CC should be measured given the findings pointing to a bifactor solution with two potential overarching measurement structures. This uncertainty could be ascribed to the study’s CC measure and items or, potentially, to deeper uncertainty regarding exact nature of the CC latent construct itself. These dual options should be viewed as a strength of the current examination, and congruent with the exploratory nature of the work herein, which *a priori* outlined multiple approaches as a sensitivity analysis.

Each measurement approach/structure has appeal and drawbacks. A general appeal of having a unidimensional CC measure is the simplicity of scoring. Also, as seen in Table 3 there are potentially meaningful questions included in the holistic CC measure that are not in the subscales. Some extant research has found support for a unidimensional conceptualization and measurement of organizational CC. For example, Zeitlin Schudrich (2014) examined the psychometric properties of an organizational CC measure using confirmatory factor analysis among child welfare agencies/providers, with results pointing to a unidimensional (i.e., 1-factor) measurement structure that included items similar to those in the measure featured in the study herein. Specifically, Zeitlin Schudrich’s final CC measure contained 6 items: (a) recruitment, hiring and retention practices; (b) representativeness of committees and councils; (c) presence of CC in monitoring and evaluation; (d) translation and interpretation; (e) appropriateness of materials; and (f) appropriateness of food (2014). Although these items are largely congruent with the items in the measure herein, and the bifactor solution suggests a possible 1-factor measure of organizational CC, drawbacks to a unidimensional approach should be considered and potentially include the lack of face validity to the notion of a single CC latent factor and loss of nuance from parsing out intra-CC factors.

Also, the bifactor solution herein suggests a second and differing multidimensional approach with multiple correlated domains within a broader CC construct. This second approach is also supported by extant research. For example, a study by Siegel et al. (2011) describes the development and evaluation of a CC scale for use in public mental health settings that included a 3-factor structure. The three factors included (a) administrative elements (e.g., commitment, staff trainings), (b) activities to understand and serve the community (e.g., gathering data, instituting recruiting/hiring/retention policies), and (c) activities directly related to clinical care (e.g., having interpreters and bilingual/bicultural staff, developing new services).

The current study determined a 4-factor bifactor model, which, to its benefit, argues for both approaches. Although similar to Zeitlin Schudrich (2014) the findings herein support a general CC factor/scale, like Siegel et al. (2011) the findings also support the notion of three subfactors/scales. The three subfactors/scales identified in the current study focus on (a) organizational values, policies, procedures, and norms; (b) cultural knowledge and inclusion; and (c) DV cultural knowledge. The first two subfactors/scales reflect broad CC related to organizational support and cultural knowledge when working with Latinx clients (Suarez-Balcazar et al., 2011). The third subfactor/scale examines knowledge regarding DV among Latinx people, including DV perceptions, experiences, needs, help-seeking, and available resources. Notably, the items in the final, reduced measure reflect domains common across other organizational CC instruments and studies including: (a) values, policies, and procedures; (b) communication; (c) community and client engagement; (d) services and service delivery; and (e) organizational resources (Cherner et al., 2014; Harper et al., 2006; Lucero et al., 2020; Zeitlin Schudrich, 2014). Ultimately, the measurement of organizational CC, and specifically within a Latinx DV service provision context, remains open for further exploration. It is likely that multiple conceptualizations and measurement approaches are valid.

### Measurement Scope

Regardless of approach, this study is clear in finding that the measure presented herein is likely associated with organizational characteristics, both converging and diverging with various variables as would be expected. Broadly these findings suggest that (a) CC as a latent construct does indeed vary across DV service providing organizations and (b) the CC measure developed herein has the ability to detect such differences.

The final measure demonstrated convergent validity as the identified factors were significantly correlated with agency characteristics theoretically expect to be related to organizational CC. Despite limited research examining the psychometric properties of organizational CC measures, research regarding DV services and service provision for Latinx survivors has highlighted the importance of providing linguistically appropriate services, hiring Latinx and Spanish-speaking staff, and engaging in culturally specific outreach (O’Neal & Beckman, 2017; Parson et al., 2016; Serrata et al., 2020)—all of which were associated with at least one of the resultant CC factors. Notably, organizational CC in the form of infrastructure and support are critical for the provision of such culturally competent and affirming practices. Further, at least three of the factors were associated with a higher percentage of staff attending CC or Latinx trainings, a higher percentage of clients that were Latinx or Spanish-speakers, and a stronger presence in and relationship with the Latinx community, all of which would be expected to be positively correlated with organizational CC. The measure also demonstrated ample discriminant validity. As expected, none of the factors were associated with whether the organization served more than one county or only rural locations, the number of full- or part-time staff at the organization, the percentage of staff that had attended DV trainings, or the total number of clients served by the organization.

Importantly, these various significant and nonsignificant associations have practical utility for intra- and inter-organizational assessment. The characteristics that demonstrated convergent validity with the CC measure could be good targets for identifying intervention targets alongside CC. These associations suggest, perhaps, that improvement on such characteristics may be associated with improvements to CC. Not every study or evaluation has the ability to ask in-depth questions of such organizations regarding their culturally competent practices. Yet, provided with basic information regarding such variables as number of services, staff case-mix, and others that could serve as proxy indicators of CC. The numerous divergent variables, meanwhile, provide further insight into what may not be important for assessing CC in this context. Overall, the measure developed herein helps to clarify the picture of organizational CC vis-à-vis Latinx DV service provision—an important contribution to an overlooked practice and research concern.

### Limitations

The study’s findings should be considered in light of several limitations. Primarily, despite the high response rate and significant buy-in from stakeholders within North Carolina, the study sample size was small for a measurement-focused analysis. Although the analyses attempted to mitigate this limitation via the use of robust analyses and multiple modeling plan, the results should be considered very much within the realm of the exploratory. This fact, coupled with the single state location, limits the external validity of the findings and perhaps the overall generalizability of the CC measure to other DV service providers in other settings. Additional and more minor limitations include the potential for the survey to not have been comprehensive in its inclusion of CC-related items, the cross-sectional nature of the data, and the lack of survivor input into the measure’s development.

### Future Research

Although there remains a need for additional research regarding CC and Latinx DV service provision, the study’s focus on measurement highlights specific foci for future examinations. To be sure, further research on the measure is likely required before widespread use can be recommended. It is also important to note that the chosen 4-factor bifactor model may not be the optimal solution. Researchers wishing to explore the similarly well-performing (a) 4-factor multidimensional or (b) 5-factor bifactor models should take the set of 29-items in Table 3 and delete items #6, #7, #12, #14, #15, and #29 to construct former, and item #24 for the latter. Data gathered on larger samples in additional settings would engender robust tests of the measure presented herein. Beyond acquiring new and more representative samples, future research should likely include analyses that seek to both (a) further refine the measure’s structure and (b) test the measure’s performance via confirmatory factor analyses and predictive validity analyses (e.g., receiver operating curve analyses) among others. All such analyses would build evidence for the validity and utility of the measure. This evidence, in turn, would work toward achieving the important distal goal of applying the measure to (a) practice-based intraorganizational assessment and (b) organizational-focused intervention and evaluation to improve CC among DV organizations serving Latinx survivors.

## Conclusion

The current study contributes to the growing literature on organizational CC by developing and evaluating a preliminary measure tailored specifically to organizational CC in the context of Latinx DV service provision in the United States. In addition, the use of bifactor EFA advances the field as this approach has heretofore been underutilized in violence measurement. Although this work is exploratory, both the general measure of CC as well as the three subfactors/scales have potential to inform the delivery and evaluation of services to Latinx DV survivors in future practice and research endeavors. Organizations can use the measure in practice to assess and enhance CC by identifying opportunities for growth. The measure can also be used in research to better understand the CC of organizations providing Latinx DV services, including the factors that impede and facilitate organizational CC as well as related client outcomes. A particular strength of this research was the centering of organizational CC specific to the delivery of services for Latinx DV survivors. By focusing directly on the measurement of CC, this work sought to echo calls for DV service provision that acknowledges the importance of cultural diversity while at the same time advancing research on measures that can help DV organizations enhance the cultural appropriateness of their services and service delivery practices. It is hoped that this study will encourage further dialogue and research regarding the measurement and understanding of organizational CC as it relates to DV service provision.

## Author Biographies

**Christopher J. Wretman**, PhD, is a senior data analyst at The Cecil G. Sheps Center for Health Services Research, and a faculty research associate at the School of Social Work, both at the University of North Carolina at Chapel Hill. His work focuses on the quantitative analysis of health and well-being outcomes to inform services for vulnerable populations.

**Cynthia Fraga Rizo**, PhD, is an assistant professor in the School of Social Work at the University of North Carolina at Chapel Hill. Her research focuses on the development and evaluation of interventions and services for addressing intimate partner violence, sexual violence, and human trafficking.

**Jeongsuk Kim**, PhD, is the Preyer Postdoctoral Scholar for Strengthening Families at the School of Social Work at the University of North Carolina at Chapel Hill. Her work focuses on a broad spectrum of interpersonal violence issues, including intimate partner violence, sexual violence, human trafficking, and school-based violence.

**Carolina Alzuru, BA,** is the senior director of the Healthier Together project at the North Carolina Counts Coalition. She has worked in the field of domestic and sexual violence prevention and advocacy services for 16 years. Carolina is particularly interested in improving victim services and prevention strategies geared toward marginalized communities.

**Deena Fulton**, MPH, is the Rape Prevention and Education Program Manager for the North Carolina Division of Public Health. She has been implementing and overseeing violence prevention and violence-related systems change work for over seven years. Deena is invested in violence prevention as social change work, particularly in exploring the connections between multiple forms of violence and transforming the social and structural dynamics that enable them to occur.

**Lisi Martinez Lotz**, PhD, is the director of planning and innovation at North Carolina Area Health Education Center. She has over 10 years of experience in the public health sector with more than 8 of those years in the domestic and sexual violence advocacy field. Her expertise includes supporting organizations in partnership building, systems change, and developing culturally appropriates services, especially for the Latinx community. At the center of Lisi’s work is health equity through the improvement of social determinants of health.
